# Leukocyte Counts, Myeloperoxidase, and Pregnancy-Associated Plasma Protein A as Biomarkers for Cardiovascular Disease: Towards a Multi-Biomarker Approach

**DOI:** 10.1155/2010/726207

**Published:** 2010-05-30

**Authors:** M. B. I. Lobbes, M. E. Kooi, E. Lutgens, A. W. Ruiters, V. Lima Passos, S. H. J. G. Braat, M. Rousch, H. Ten Cate, J. M. A. van Engelshoven, M. J. A. P. Daemen, S. Heeneman

**Affiliations:** ^1^Department of Pathology, Cardiovascular Research Institute Maastricht, P.O. Box 5800, 6202 AZ Maastricht, The Netherlands; ^2^Department of Radiology, Maastricht University Medical Center, P.O. Box 5800, 6202 AZ Maastricht, The Netherlands; ^3^Department of Cardiology, Orbis Medical Center, P.O. Box 5500, 6130 MB Sittard, The Netherlands; ^4^Department of Methodology and Statistics, Maastricht University, P.O. Box 616, 6200 MD Maastricht, The Netherlands; ^5^Department of Cardiology, Maastricht University Medical Center, P.O. Box 5800, 6202 AZ Maastricht, The Netherlands; ^6^Department of Internal Medicine, Maastricht University Medical Center, P.O. Box 5800, 6202 AZ Maastricht, The Netherlands

## Abstract

We evaluated leukocyte counts and levels of CRP, fibrinogen, MPO, and PAPP-A in patients with stable and unstable angina pectoris, acute myocardial infarction, and healthy controls. All biomarkers were analyzed again after 6 months. Leukocyte counts and concentrations of fibrinogen, CRP, MPO, and PAPP-A were significantly increased in patients with acute myocardial infarction. Leukocyte counts and concentrations of MPO were significantly increased in patients with unstable angina pectoris compared with controls. After 6 months, leukocyte counts and MPO concentrations were still increased in patients with acute myocardial infarction when compared to controls. Discriminant analysis showed that leukocyte counts, MPO, and PAPP-A concentrations classified study group designation for acute coronary events correctly in 83% of the cases. In conclusion, combined assessment of leukocyte counts, MPO, and PAPP-A was able to correctly classify acute coronary events, suggesting that this could be a promising panel for a multibiomarker approach to assess cardiovascular risk.

## 1. Introduction

Atherosclerosis is an inflammatory disease of the large arteries that is characterized by the formation of atherosclerotic plaques. In the majority of cases, atherosclerosis-related clinical events, like myocardial infarction or ischemic stroke, are caused by rupture of a vulnerable atherosclerotic lesion [1–3]. 

Several inflammatory molecules have been put forward as biomarkers for plaque vulnerability. Biomarkers are biochemical features that can be used to measure the presence of a certain disease, the disease progress, or the effect of treatment [4]. In the context of atherosclerosis, concentrations of C-reactive protein (CRP) and fibrinogen and the count of leukocytes in blood have been investigated most extensively [5–7]. However, large meta-analyses have demonstrated that their prognostic value for assessing risk of cardiovascular disease or adverse outcomes is limited [7–10]. Therefore, there is a continuous search for novel, more powerful biomarkers that are able to predict the occurrence of future cardiovascular complications. 

Until now, no single biomarker has been able to accurately predict the risk of near-future cardiovascular events in the individual patient. The general opinion is therefore shifting towards a so-called “multi-biomarker” approach, in which a certain panel of biomarkers is assessed to determine an individual risk profile of a patient for cardiovascular disease [4]. However, it remains unclear which biomarkers should be included in this panel.

Our study aim was to assess levels of selected biomarkers simultaneously in several groups of patients with cardiovascular disease. We focused on leukocyte counts and concentrations of fibrinogen, CRP, MPO, and PAPP-A, as these biomarkers have been studied extensively in large cohorts (leukocyte counts, fibrinogen, CRP [7–10]) or have shown potential in smaller cohorts (MPO, PAPP-A [11–13]). In addition, we investigated the levels of these five biomarkers after 6-month followup to evaluate changes in this period, and we used a stepwise discriminate analysis to investigate which of the currently tested biomarkers might be most appropriate to include in a “multi-biomarker” panel.

## 2. Materials and Methods

For this study, the total study cohort consisted of 120 patients, who were divided into four study groups: stable angina pectoris (SAP), unstable angina pectoris (UAP), acute myocardial infarction (AMI), and healthy controls (CON). Venous blood samples were drawn from all participants at inclusion and after 6-month followup. Also, a standardized questionnaire regarding patient characteristics, risk factors, and followup outcome (such as the occurrence of new or recurrent clinical events) was presented at study inclusion and followup. Medication use was assessed during study inclusion and included beta-blockers, oral nitrates, ACE inhibitors, statins, fibrates, calciumantagonists, insulin, aspirin, hormone replacement therapy, and antidiabetics. The study protocol was approved by the institutional medical ethics committee. All patients gave written, informed consent prior to study inclusion. The funders had no role in study design, data collection and analysis, decision to publish, or preparation of the manuscript.

Patients with SAP that were scheduled for a percutaneous coronary intervention were recruited from the outpatient clinic. Only patients with more than 50% stenosis of one or more of the main coronary branches (as proven by coronary angiography) were included. Evaluation of the coronary stenosis was performed by cardiologists blinded for study aims. Patients with UAP presented themselves with prolonged new-onset chest pain (<30 days), an accelerating pattern of chest pains or with chest pains occurring at lesser degrees of exertion or at rest. UAP was characterized by ischemic ECG changes (such as ST segment elevation, reciprocal ST segment depression, T wave inversion, or development of Q waves) without elevation of cardiac enzymes (such as troponin-T, creatine kinase, and lactate dehydrogenase isozymes) or by elevation of cardiac enzymes without apparent ischemic ECG changes. Patients with AMI presented themselves with an acute onset of chest pain, ischemic ECG changes, and elevation of cardiac enzymes and troponin T. Diagnoses of UAP or AMI were made by cardiologists blinded for the study aims. The control group consisted of individuals of the general population older than 45 years, but free of cardiovascular disease and serious illnesses for the past 6-month. 

In all groups, exclusion criteria were inability to provide informed consent, recent acute coronary event, stroke or transient ischemic attack (all events less than 6-month prior to study inclusion), history of resuscitation or cardiogenic shock, renal insufficiency (creatinine clearance <40 mL/min according to the Cockroft formula [14]), current inflammatory disease, autoimmune diseases, and the presence of a disorder with a high chance of death within 5 years (e.g., malignancies).

Blood samples were drawn from the antecubal vein. In patients with SAP, blood sampling was performed immediately prior to percutaneous coronary intervention. In patients with UAP and AMI, blood was drawn and processed within 12 hours after the last ischemic episode and before intervention. Levels of fibrinogen, total cholesterol, HDL-C, LDL-C, liver function enzymes (AST, ALT), creatine kinase, creatinine, and leukocyte counts were determined with standard hospital laboratory assays. The blood samples used for the determination of CRP, PAPP-A, and MPO were centrifuged immediately after sampling and frozen at −80°C until assays were performed. All samples were thawed only once. By using coded samples, all laboratory analyses were performed blinded for study group designation.

Enzyme-linked immunosorbent assays (ELISA) were performed to determine serum levels of CRP (Kordia, Leiden, The Netherlands), PAPP-A, and MPO (all DRG Instruments GmbH, Germany). In addition, a new ultrasensitive assay kit for PAPP-A (PAPP-A US) was used. This was a research kit from DRG Instruments, which uses a monoclonal antibody specific for patients with AMI, whereas the regular kit uses a polyclonal antibody. Assays were performed according to the instructions provided by the manufacturer. Assay ranges were 1–25 mg/L for CRP, 0–30 *μ*g/l for PAPP-A, and 0–450 *μ*g/L for ultra-sensitive PAPP-A. For MPO, the detection limit was <3 *μ*g/L.

### 2.1. Statistical Analysis

Statistical analysis was carried out with SPSS 15.0 (Chicago, USA). Since the distribution of biomarker levels was slightly skewed, statistical tests were performed on logarithmically transformed biomarker values, while mean (arithmetic) values of these values were presented. Depending on the research questions and the outcome variables, three different tests were applied. First, an univariate analysis of variance (ANOVA) was used to determine which variables were significantly associated with the biomarkers baseline measurements. Multicollinearity among explanatory variables (see also Table 1) was checked via the variance inflation factor and a top-down procedure was used to determine the final model parameters. For all models, the group's effect on the individual biomarkers was adjusted for sex, smoking, family history of cardiovascular disease, daily aspirin use, and exercise (defined as a significant increase in heart rate for more than 30 minutes per week), since these proved to be relevant parameters that needed to be included in the final model. Additional adjustment for age and renal clearance was performed in the analysis of fibrinogen and PAPP-A concentrations, and for body mass index in the analysis of CRP. All other patient characteristics as noted in the questionnaires proved to be nonrelevant model parameters and as such, were not included in the statistical analysis. Adjusted post-hoc groups comparisons were carried out using the Bonferroni correction for multiple comparisons. Second, a repeated measures ANOVA was used in order to evaluate changes in biomarker levels in time with groups as a fixed between-subjects factor. Third, a stepwise discriminant analysis was performed to assess which biomarkers could best discriminate between the four study groups. All *P*-values ≤.05 were considered statistically significant.

## 3. Results

During followup, no patients were lost for analysis. Only two patients suffered from an acute myocardial infarction during the followup period: one UAP, one AMI. General patient characteristics are presented in Table 1. Gender, smoking, exercise, family history for cardiovascular disease, the use of beta-blockers, aspirin and statins, creatine kinase, troponin T, cholesterol and LDL-C levels, and diastolic blood pressure differed significantly between study groups. In addition, it is known that the concentrations of different biomarkers can be highly dependent on the time between the onset of symptoms and sample collection [12]. Nonetheless, the mean time from symptoms to sample collection did not differ significantly in the acute coronary events study groups (UAP and AMI): 7.8 (range 2.0–10.9) and 6.9 (range 1.2–11.3) hours, respectively.

**Figure 1:**
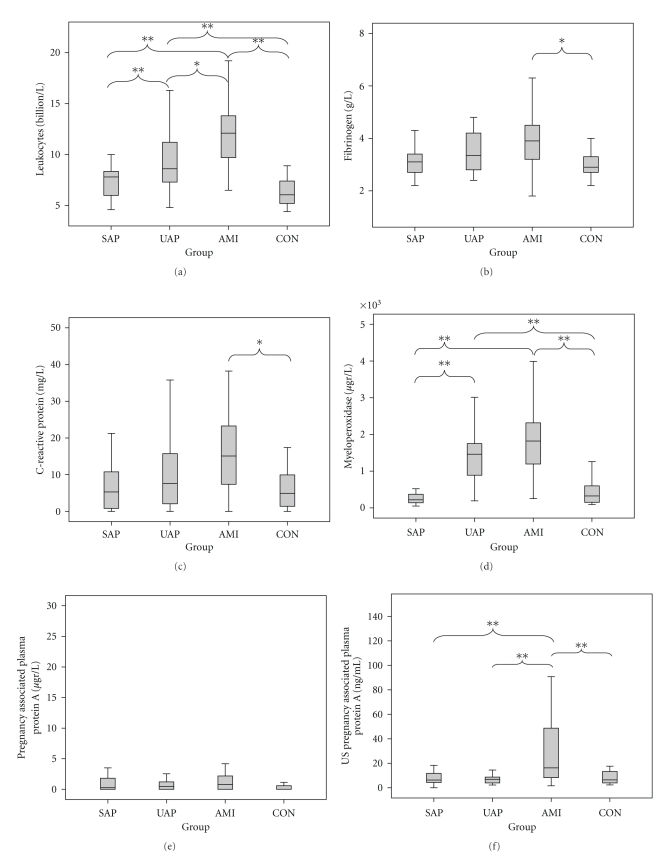
Boxplots of biomarkers levels at study inclusion of leukocyte counts (a), fibrinogen (b), CRP (c), myeloperoxidase (d), PAPP-A (e), and US PAPP-A (f). Statistical differences are expressed as *P* < .05 (*) ot *P* ≤ .001 (**).

**Figure 2:**
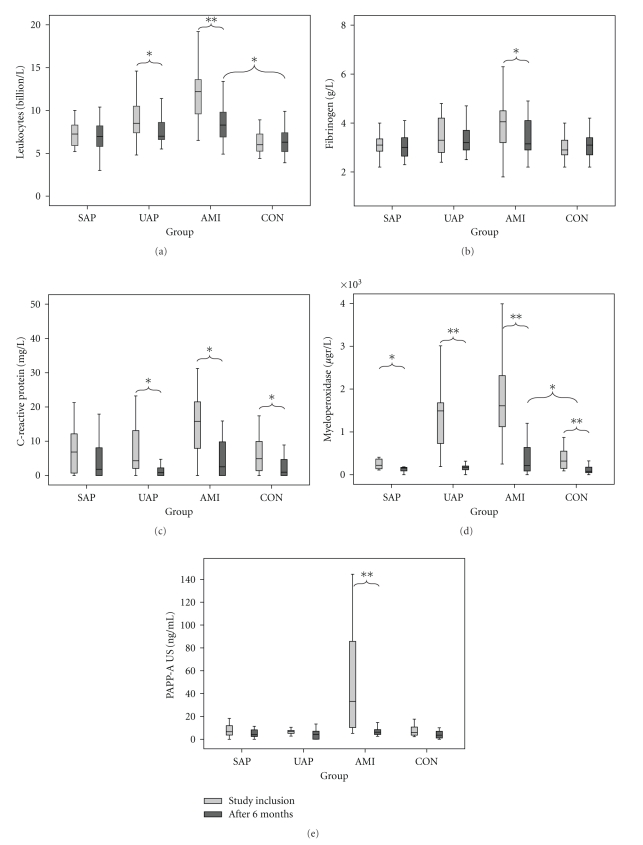
Boxplots of biomarker levels of leukocyte counts (a), fibrinogen (b), C-reactive protein (c), myeloperoxidase (d), and ultrasensitive pregnancy-associated plasma protein A (e) at study inclusion and after 6-month followup. Statistical differences at 6-month followup were expressed as *P* < .05 (*) or *P* ≤ .001 (**).

At study inclusion, leukocyte counts differed significantly between controls and patients with AMI or UAP (both *P* < .001). There was no significant difference between controls and patients with SAP. In addition, leukocyte counts were significantly higher in patients with AMI than in patients with UAP (*P* = .002) or SAP (*P* < .001, Figure 1 and Table 2). There was a significant decrease in leukocyte counts for patients with UAP or AMI after 6-months (*P* = .011 and *P* < .001, resp.), while there were no significant changes in leukocyte counts in patients with SAP or controls. A significantly increased leukocyte count between AMI and CON remained (*P* = .002), whereas all other pairwise comparisons were nonsignificant (Figure 2).

For fibrinogen, serum concentrations were significantly higher in patients with AMI compared with controls (*P* = .002). There was no significant difference between CON and UAP or SAP, or between AMI and UAP, or between UAP and SAP (Figure 1 and Table 2). However, there was a trend for a difference between AMI and SAP (*P* = .07). After 6-month followup, there was only a significant decrease in fibrinogen concentration in patients with AMI (*P* = .034). There were no significant differences in fibrinogen concentrations between different study groups at this time point (Figure 2).

For CRP, there was a significantly higher concentration in patients with AMI compared with controls (*P* = .02). There was no significant difference between controls and patients with UAP or SAP, and no significant differences between AMI and SAP or UAP, or between UAP and SAP (Figure 1, Table 2). At followup, CRP concentrations were lower in patients with UAP (*P* = .009) or AMI (*P* = .009), and CON (*P* = .016). At this time point, there were no significant differences in CRP concentrations between different patient groups (Figure 2).

For MPO, serum concentrations were significantly higher in patients with UAP (*P* < .001) or AMI (*P* < .001) compared with controls. There was no significant difference in serum MPO concentrations between patients with SAP or controls. Furthermore, serum MPO levels were significantly higher in AMI and UAP compared with SAP (both *P* < .001), but there was no difference between AMI and UAP (Figure 1 and Table 2). At followup, the mean MPO concentrations had significantly decreased in patients with SAP (*P* = .008), UAP (*P* < .001), or AMI (*P* < .001) and CON (*P* < .001). At 6-month, MPO concentrations in patients with AMI were still increased when compared with CON (Figure 2, *P* = .046).

Finally, for PAPP-A, no significant differences were demonstrated between the controls and the three groups of cardiovascular disease for the regular assay kit (Figure 1, Table 2). Based on these results, we decided not to analyze serum PAPP-A concentrations in the 6-month followup blood samples with the regular assay kit. In contrast, the US PAPP-A kit showed a significantly higher concentration of PAPP-A in AMI when compared with the other study groups (all *P* = .001). After 6-month, PAPP-A concentrations of AMI returned to baseline values (*P* < .001) and no significant differences in PAPP-A concentrations could be observed between study groups (Figure 2).

A stepwise discriminant analysis was performed to investigate which combination of the five biomarkers could differentiate between the four groups at study inclusion. In the stepwise procedure, only leukocyte counts, MPO, and PAPP-A US emerged as having significant discriminative power. Based on linear combinations of these markers, subjects could be properly allocated to their specific groups in 61.6% (58.6% in the leave-one-out cross-validation) of the cases (Table 3). Visual inspection of the discriminant functions' territory map revealed that the centroids of SAP and CON almost overlapped, suggesting similarity of profiles as regards their biomarkers' distributions. This motivated three subsequent analyses. In the first one, SAP and CON were combined in one group. With this new subdivision (i.e., three diagnostic categories), patients' correct classification improved to 82.8% (cross-validation 80.8%), based on the same three biomarkers (Table 3). In the second analysis, a broader dichotomous categorisation of patients in nonacute coronary events/controls (SAP and CON) versus acute coronary events (UAP and AMI) was considered. Here, patients' classification by leukocyte counts and MPO was correct in 89.3% of the cases (cross-validation 87.5%, Table 3). However, PAPP-A US had no further contribution and the discriminant functions were based solely on leukocyte counts and MPO. In contrast, PAPP-A US and leukocyte counts were the only markers selected in the third analysis, in which discriminant functions were required to separate between AMI and UAP patients. In this last analysis, 82.4% of the acute patients were correctly assigned to their corresponding groups (cross-validation 80.4%, Table 3).

## 4. Discussion

In the current study, we evaluated leukocyte counts and levels of CRP, fibrinogen, MPO, and PAPP-A in patients with stable (SAP) and unstable angina pectoris (UAP), acute myocardial infarction (AMI), and healthy controls (CON). These obtained results showed that inflammatory markers were increased in patients with acute coronary events (especially myocardial infarction) and were in line with available literature, which also showed an increase of these markers after acute coronary events [9, 11, 15]. Followup analysis of these biomarkers after 6-month showed in general a significant decrease in serum concentrations of these biomarkers in acute coronary syndromes, returning to concentrations comparable with those of SAP and CON. However, after six months, leukocyte counts and serum concentrations of MPO remained significantly higher in patients with AMI when compared with controls. A discriminant analysis showed that of these five tested biomarkers, leukocyte counts, MPO, and PAPP-A were most accurate in predicting study group designation.

Many studies have highlighted the predictive value of CRP for cardiovascular events. However, large meta-analyses showed only moderate prognostic value (relative risk estimates in the range of 1.3 to 1.5) [7, 9]. Similar results have been published on the use of fibrinogen concentrations and leukocyte counts for the prediction of cardiovascular events and outcome (relative risk estimates of 1.4 to 1.8 for fibrinogen and approximately 1.4 for leukocyte counts) [7, 15, 16]. This creates a need for additional biomarkers for cardiovascular disease. With this respect, MPO and PAPP-A have been studied for their potential to serve as novel biomarkers for cardiovascular disease [11, 13, 17].

MPO has been associated with cardiovascular disease in many studies [18]. Zhang et al. showed that elevated serum levels of MPO were associated with coronary artery disease, as visualized by angiography [19]. These results are in line with our observations, since we found a significant increase in serum MPO levels in patients with UAP or AMI, when compared with healthy controls. Additional studies demonstrated an increased cardiac risk with elevated serum levels of MPO in patients with acute coronary syndromes, and an increased odds ratio for major adverse cardiac events in a cohort of patients presenting with chest pains [11, 20–22]. Above median plasma, MPO concentrations of AMI patients were associated with an increased risk for mortality [23]. In patients with chest pain, MPO proved to be an independent predictor of AMI on long-term followup [24]. We did not find any significant difference in MPO levels between controls and patients with SAP, which is in contrast to data published by Ruef et al., that showed an increased concentration of plasma MPO levels for patients with SAP or acute coronary syndromes [25]. However, direct comparison of MPO levels between studies remains difficult, because many studies differ in their blood sample collection (plasma or serum) and their laboratory assays to determine MPO concentrations [18].

Positive immunohistochemical staining for PAPP-A in advanced atherosclerotic plaques and an elevation of PAPP-A levels in acute coronary syndromes were shown by Bayes-Genis et al. [17]. PAPP-A is an independent predictor of adverse outcome, and elevated levels were associated with an increased risk for death or myocardial infarction in patients with acute or stable coronary syndromes [26–28]. In line with our present findings, Dominguez-Rodriguez et al., applying the regular PAPP-A kit as used in the present study (DRG International), did not find any significant differences in PAPP-A concentrations between patient with AMI and controls, and no association between PAPP-A levels and risk of AMI [29]. However, we currently showed that the ultrasensitive PAPP-A research kit, using a monoclonal antibody specific for patients with AMI, did show a significantly higher concentration of PAPP-A in AMI when compared with controls. In healthy controls, PAPP-A is present in forms complexed with or without the proform of the eosinophilic major basic protein (proMBP). Uncomplexed PAPP-A originates from advanced atherosclerotic lesions [30], and is most likely produced by vascular endothelial and smooth muscle cells [17]. However, current commercially available ELISA kits for the detection of PAPP-A are designed to detect Down's syndrome and therefore focus on the total amount of PAPP-A present in serum (both complexed and uncomplexed with proMBP). In order to accurately assess the association and predictive value of PAPP-A levels in patients with acute coronary syndromes, the levels of uncomplexed PAPP-A need to be determined. This might explain the different results that were obtained between studies with custom made assays and commercially available assays. Future studies should focus on the association of this uncomplexed PAPP-A variant and cardiovascular disease [31]. In the current study, we demonstrated a significantly higher concentration of PAPP-A in AMI when compared with the other study groups using the ultrasensitive kit. After 6-month, the PAPP-A concentrations of AMI dropped to baseline values and no differences in PAPP-A concentrations between different patient groups could be observed. Therefore, this ultrasensitive PAPP-A kit might aid in the detection of atherosclerosis-specific PAPP-A.

Although individual biomarker concentration values may in average show significant increase for cardiovascular patients, the biomarkers' variances are generally still rather higher, resulting in clearly lower biomarker levels in several affected individuals. Therefore, it is currently believed that it is unlikely that a *single* biomarker will be discovered for predicting cardiovascular events. The general opinion is shifting towards a multimarker approach, in which a certain panel of biomarkers is assessed to determine an individual risk profile of a patient for cardiovascular disease [4, 32]. For example, Varo et al. demonstrated that patients with elevated levels of both soluble CD40 ligand and troponin showed a marked increase in adjusted hazard ratio (HR) for adverse outcomes (HR 4.3) when compared with soluble CD40 ligand alone (HR 1.9) [33]. Ardigo et al. demonstrated that a combination of serum levels of multiple chemokines (such as CCL2, CCL7, CCL8, CCL13, and CXCL10) resulted in a high degree of accuracy in predicting clinically significant atherosclerotic heart disease in patients with and without clinically manifest coronary artery disease [34]. In addition, Wang et al. measured ten different biomarkers (CRP, B-type natriuretic peptide, N-terminal proartrial natriuretic peptide, aldosterone, rennin, fibrinogen, D-dimer, plasminogen-activator inhibitor-1, homocysteine, and urinary albumin-to-creatinine-ratio) in 3,209 participants of the Framingham Heart Study and monitored the participants for a followup period of 7.4 years [32]. Participants with high multi-marker levels had a four times increased risk of death and a twofold increased risk of major cardiovascular events when compared with participants with low multi-marker levels.

However, it remains unclear which biomarkers should be included in this panel. With this respect, a stepwise discriminant analysis of our data of the five tested biomarkers showed that leukocyte counts and MPO were powerful biomarkers, with regard to the accurate distinction between patients who had acute coronary events from those who did not (Table 3). Slightly less accurate but still reasonably high was the discrimination between AMI, UAP, and a third group that combined SAP and CON. This time PAPP-A US contributed significantly to the other two biomarkers (leukocyte counts and MPO). Moreover, within the acute patients only (i.e., UAP and AMI), PAPP-A US showed a significant discriminative power, in combination with leukocyte counts. By contrast, once all four original diagnostic groups were considered, the classification of patients according to these three biomarkers was suboptimal as only 62% of the cases were classified correctly (Table 3). Thus, healthy controls and patients with SAP share similar biomarkers profiles, indicating that their clear-cut separation would require additional diagnostic criteria. 

These findings of this discriminant analysis suggest that of the biomarkers tested in this study, leukocyte counts, MPO, and PAPP-A US have the most potential as biomarkers of acute cardiovascular disease and might be suitable candidates to be included in the ‘multi-biomarker approach' to assess the cardiovascular risk. Future studies should assess the predictive value of this multi-marker approach for cardiovascular events. We additionally want to emphasize that the clinical application of a multi-marker approach depends not only on using the most promising biomarkers, but also on the ratio between costs and benefits. Therefore, this should also be taken into account in future studies. 

In conclusion, leukocyte counts and levels of fibrinogen, CRP, myeloperoxidase, and pregnancy associated plasma protein-A were increased in patients with acute coronary syndromes. In addition, a discriminant analysis of the five biomarkers currently tested showed that leukocyte counts, MPO, and PAPP-A were most powerful in accurately assessing study group designation, suggesting that this could be a promising panel for a multi-biomarker approach to assess cardiovascular risk.

## Figures and Tables

**Table 1:** Baseline patient characteristics per study group.

	SAP	UAP	AMI	CON	*P*-value
Total participants	30	30	30	30	
Age (mean years ± SD)	63.6 (10.5)	65.3 (13.0)	59.5 (12.6)	65.0 (9.5)	NS
Male (%)	28 (93.3)	16 (55.1)	24 (80.0)	19 (63.3)	.004
Smoking (%)	7 (23.3)	5 (16.7)	13 (43.3)	4 (13.3)	.034
Exercise (%)	14 (46.7)	15 (50.0)	8 (26.7)	22 (73.3)	.004
Alcohol use (U/day ± SD)	0.7 (1.3)	0.4 (0.7)	0.5 (0.9)	1.3 (1.1)	NS
Family history for CVD (%)	26 (86.7)	18 (60.0)	14 (46.7)	19 (63.3)	.013
Diabetes (%)	3 (10.0)	7 (23.3)	1 (3.3)	3 (10.0)	NS
Beta-blocker use (%)	30 (100.0)	15 (50.0)	12 (40.0)	5 (16.7)	<.001
Statin use (%)	26 (86.7)	18 (60.0)	12 (40.0)	6 (20.0)	<.001
Aspirin use (%)	13 (43.3)	14 (46.7)	12 (40.0)	1 (3.3)	.001
Antidiabetica use (%)	2 (6.7)	5 (16.7)	1 (3.3)	3 (10.0)	NS
Cholesterol level (mmol/L ± SD)	4.2 (1.0)	4.7 (1.3)	5.5 (1.1)	5.6 (1.3)	<.001
HDL-C (mmol/L ± SD)	1.1 (0.2)	1.0 (0.2)	1.0 (0.2)	1.2 (0.4)	NS
LDL-C (mmol/L ± SD)	2.1 (1.0)	3.0 (1.1)	3.9 (1.0)	3.6 (1.1)	<.001
ALT (IU/L± SD)	34 (17)	31 (19)	37 (22)	26 (29)	NS
AST (IU/L± SD)	26 (13)	30 (16)	99 (139)	22 (11)	NS
Renal clearance (mL/min ± SD)	85.8 (29.7)	83.7 (28.2)	93.2 (20.1)	83.7 (26.4)	NS
Systolic BP (mmHg ± SD)	131.0 (14.8)	141.3 (22.0)	129.7 (30.0)	132.2 (15.6)	NS
Diastolic BP (mmHg ± SD)	76.6 (7.9)	77.5 (15.3)	73.8 (15.4)	82.8 (8.6)	.001
Body mass index (kg/m^2^± SD)	26.4 (3.7)	25.8 (2.9)	27.1 (3.5)	26.4 (3.9)	NS
Troponin-T (*μ*g/L ± SD)	0.00 (0.00)	0.21 (0.13)	2.08 (0.73)	0.00 (0.00)	.003
Creatine kinase (U/L)	154.0 (107.5)	148.3 (141.3)	639.6 (913.8)	116.1 (47.4)	<.001

Exercise is defined as a significant rise in heart rate for more than 30 minutes and at least once a week. Abbreviations: (NS) nonsignificant parameters, standard deviation (SD), units (U), cardiovascular disease (CVD), and blood pressure (BP).

**Table 2:** Pairwise comparisons of serum biomarker level differences.

Leukocyte count (billions/L)				Fibrinogen (g/L)			
Group	Comp.	Mean Δ	*P*-value	Group	Comp.	Mean Δ	*P*-value
CON	SAP	0.246	NS	CON	SAP	0.052	NS
	UAP	2.485	<.001		UAP	0.236	NS
	AMI	4.980	<.001		AMI	0.712	.02
AMI	SAP	4.733	<.001	AMI	SAP	0.764	NS
	UAP	2.495	.002		UAP	0.476	NS
UAP	SAP	2.239	<.001	UAP	SAP	0.288	NS

C-reactive protein (mg/L)				Myeloperoxidase (*μ*g/L)			
Group	Comp.	Mean Δ	*P*-value	Group	Comp.	Mean Δ	*P*-value

CON	SAP	1.428	NS	CON	SAP	86.59	NS
	UAP	1.408	NS		UAP	986.55	<.001
	AMI	9.505	.02		AMI	1375.86	<.001
AMI	SAP	8.077	NS	AMI	SAP	1462.44	<.001
	UAP	8.097	NS		UAP	398.31	NS
UAP	SAP	0.020	NS	UAP	SAP	1073.14	<.001

PAPP-A (mg/L)				US PAPP-A (ng/mL)			
Group	Comp.	Mean Δ	*P*-value	Group	Comp.	Mean Δ	*P*-value

CON	SAP	0.080	NS	CON	SAP	1.288	NS
	UAP	0.187	NS		UAP	2.123	NS
	AMI	0.129	NS		AMI	25.527	.001
AMI	SAP	0.050	NS	AMI	SAP	32.885	.001
	UAP	0.060	NS		UAP	54.200	.001
UAP	SAP	0.110	NS	UAP	SAP	1.648	NS

Pairwise comparisons of different biomarker levels between study groups. Abbrevation: not significant (NS).

**Table 3:** Results of different discriminant analyses.

Groups (total number)	Significant biomarkers	Correct classification
CON-SAP-UAP-AMI (4)	Leukocyte counts, MPO, PAPP-A US	61.6%
CON/SAP-UAP-AMI (3)	Leukocyte counts, MPO, PAPP-US	82.8%
CON/SAP-UAP/AMI (2)	Leukocyte counts, MPO	89.3%
UAP-AMI (2)	Leukocyte counts, PAPP-A US	82.4%
